# The Origin of the *RB1* Imprint

**DOI:** 10.1371/journal.pone.0081502

**Published:** 2013-11-25

**Authors:** Deniz Kanber, Karin Buiting, Christian Roos, Jörg Gromoll, Sabine Kaya, Bernhard Horsthemke, Dietmar Lohmann

**Affiliations:** 1 Institut für Humangenetik, Universitätsklinikum Essen, Essen, Germany; 2 Gene Bank of Primates und Abteilung Primatengenetik, Deutsches Primatenzentrum, Leibniz-Institut für Primatenforschung, Göttingen, Germany; 3 Centrum für Reproduktionsmedizin und Andrologie, Universitätsklinikum Münster, Münster, Germany; University of Florence, Italy

## Abstract

The human *RB1* gene is imprinted due to a differentially methylated CpG island in intron 2. This CpG island is part of *PPP1R26P1*, a truncated retrocopy of *PPP1R26*, and serves as a promoter for an alternative *RB1* transcript. We show here by *in silico* analyses that the parental *PPP1R26* gene is present in the analysed members of Haplorrhini, which comprise Catarrhini (Old World Monkeys, Small apes, Great Apes and Human), Platyrrhini (New World Monkeys) and tarsier, and Strepsirrhini (galago). Interestingly, we detected the retrocopy, *PPP1R26P1*, in all Anthropoidea (Catarrhini and Platyrrhini) that we studied but not in tarsier or galago. Additional retrocopies are present in human and chimpanzee on chromosome 22, but their distinct composition indicates that they are the result of independent retrotransposition events. Chimpanzee and marmoset have further retrocopies on chromosome 8 and chromosome 4, respectively. To examine the origin of the *RB1* imprint, we compared the methylation patterns of the parental *PPP1R26* gene and its retrocopies in different primates (human, chimpanzee, orangutan, rhesus macaque, marmoset and galago). Methylation analysis by deep bisulfite sequencing showed that *PPP1R26* is methylated whereas the retrocopy in *RB1* intron 2 is differentially methylated in all primates studied. All other retrocopies are fully methylated, except for the additional retrocopy on marmoset chromosome 4, which is also differentially methylated. Using an informative SNP for the methylation analysis in marmoset, we could show that the differential methylation pattern of the retrocopy on chromosome 4 is allele-specific. We conclude that the epigenetic fate of a *PPP1R26* retrocopy after integration depends on the DNA sequence and selective forces at the integration site.

## Introduction

Retrotransposition describes the process of reverse-transcription of an mRNA followed by the insertion into a new genomic location, thus forming a retrocopy of its parental gene [1]. This mechanism provides a source for genome variability and contributes to genome evolution. Retrocopies were misleadingly thought to be “junk DNA” for a long time as they do not have promoter regions and often carry deletions or mutations, but many functional retrocopies have been detected in the past years. Functional retrocopies can be either protein-coding [2] or they can have a regulatory function as they can serve as miRNA decoys, produce siRNAs or function as antisense transcripts [3,4,5]. *Mcts2*, *Nap1l5* and *Zrsr1* are imprinted retrocopies located in introns that influence the expression of their respective host genes by transcriptional interference [6,7,8,9]. Epigenetically controlled transcriptional interference by retrocopies also takes place on human chromosome 13, where intron 2 of the *RB1* gene harbours *PPP1R26P1*, a truncated retrocopy of *PPP1R26*. In contrast to the aforementioned retrogenes, a fusion of the retrocopy and the host gene has developed at this locus. *PPP1R26P1* is integrated in reverse orientation relative to *RB1* and its exon 4 contains a CpG island that gained promoter activity and imprinted methylation. Moreover, this CpG island harbours a start exon which is spliced onto exon 3 of the *RB1* gene leading to skewed *RB1* expression [10]. Because mouse *Rb1* neither contains the retrocopy nor shows imprinted expression, we focused our analyses on primates. We compared the sequences of the homologous retrocopies in the available primate genomes, determined methylation patterns of the included CpG islands and identified expression of transcripts initiating within these regions to examine the origin of the human *RB1* imprint.

## Results

### Primate genomes contain retrocopies of *PPP1R26*

A Blat search with the mRNA sequence of the *PPP1R26* gene showed that the genomes available from Anthropoidea (human - *Homo sapiens*, chimpanzee - *Pan troglodytes*, orangutan - *Pongo abelii*, rhesus macaque - *Macaca mulatta*, marmoset - *Callithrix jacchus*), tarsier (*Tarsius syrichta*) and galago (*Otolemur garnettii*) contain this gene with conserved exon-intron organization. We could not determine if the 5'-part of *PPP1R26* is conserved in the following species as the genome information available for rhesus macaque, marmoset and galago showed sequencing gaps in the regions corresponding to exon 1, and the tarsier genome data is incomplete so only part of exon 4 of *PPP1R26* was detected. We conclude that *PPP1R26* is present in tarsier although only partly detected because of the presence of *PPP1R26* in Anthropoidea as well as in galago.

**Figure 1:**
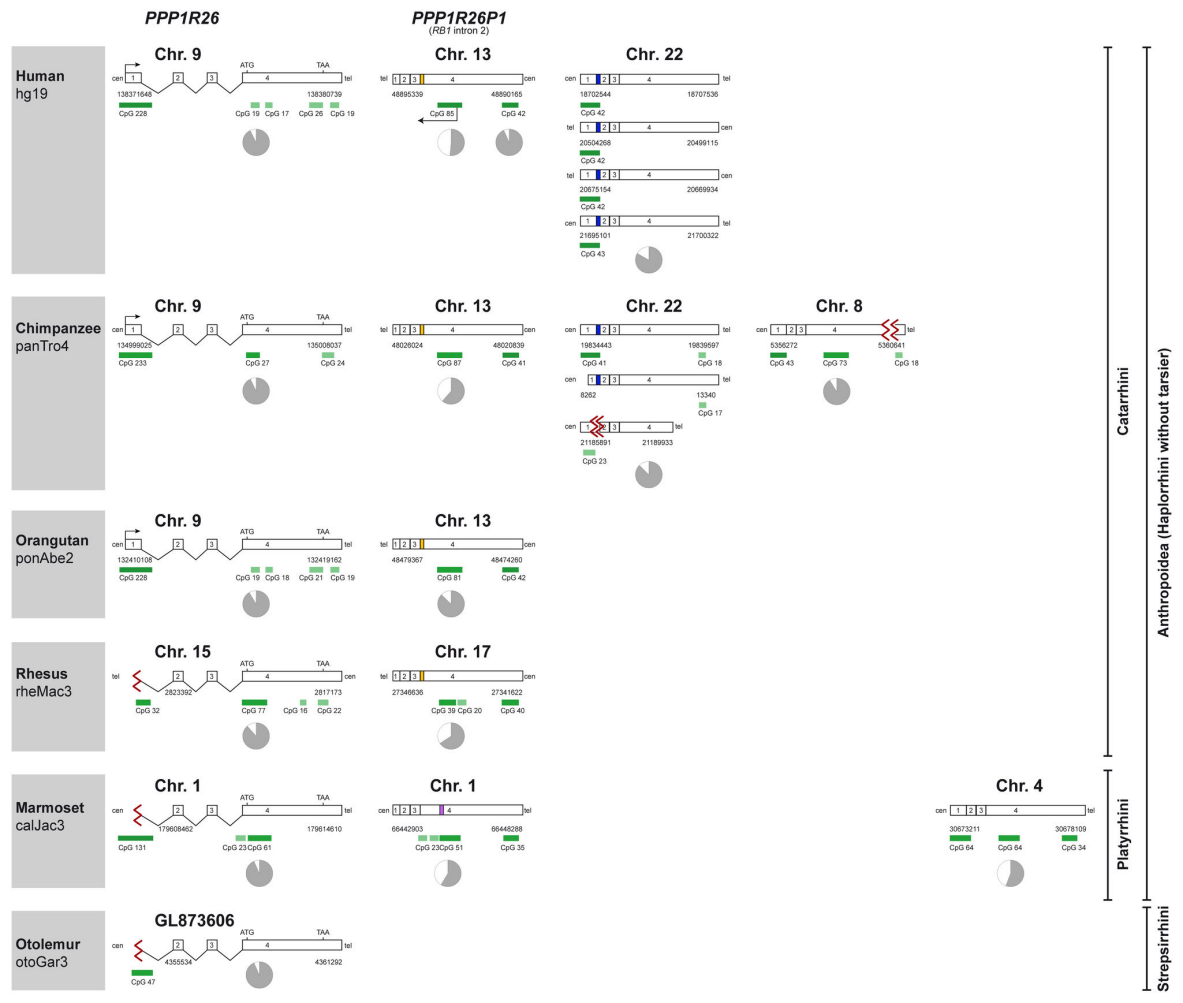
Primate genomes contain retrocopies of *PPP1R26*. All primates analysed contain the *PPP1R26* gene. All Anthropoidea have *PPP1R26P1*, a 5’-truncated retrocopy of *PPP1R26* inserted into intron 2 of the RB1 gene, which is missing in galago (*Otolemur garnettii*, Strepsirrhini). All Anthropoidea except marmoset have an Alu element (yellow box) inserted in this retrocopy. Apart from the retrocopies in the RB1 gene, the Blat search revealed additional retrocopies on chromosome 22 in human (four retrocopies) and chimpanzee (three retrocopies) and one additional retrocopy on chimpanzee chromosome 8 and marmoset chromosome 4. The pie charts indicate the position of the analysed CpG islands and their degree of methylation (grey, methylated; white, unmethylated). yellow box, Alu element; purple box, ~550 bp duplication; blue box, ~250 bp intronic sequence of intron 1 of the parental gene; green boxes, CpG islands (>300 bp dark; <300 bp light); red sigma sign, gap.

The Blat search revealed *PPP1R26P1*, a 5’-truncated retrocopy of *PPP1R26*, in intron 2 of the *RB1* gene in all Anthropoidea studied (Figure 1). However, two copies of *PPP1R26P1* are annotated in the genome database of orangutan in *RB1* intron 2 as head-to-tail tandem repeats. This annotation could be incorrect as these two copies represent end sequences of neighbouring sequence contigs with a sequence gap between the contigs. This was supported by the results of our quantitative PCR that showed only one copy of *PPP1R26P1* in orangutan (Figure S1). *PPP1R26P1* was found neither in tarsier nor in galago, irrespective of which tool (Blat, blastn or Progressive Mauve) was used (Figure S2). The retrocopies in intron 2 of the *RB1* gene in human, chimpanzee, orangutan and rhesus macaque genomes have an Alu repeat insertion of about 300 bp between the regions corresponding to exon 3 and exon 4 of the *PPP1R26* gene, but the insertion has no sequence identity to *PPP1R26*. The marmoset *PPP1R26P1* has no Alu insertion but a duplication of about 530 bp in the region corresponding to exon 4 of *PPP1R26*.

On chromosome 22 of human and chimpanzee there are further 4 and 3 retrocopies, respectively. In human, all chromosome 22 retrocopies consist of the complete exons 1, 2, 3 and 4 and have an insertion of about 250 bp between exon 1 and exon 2 of the parental sequences that has 81% sequence identity to the 5'-region of intron 1 of *PPP1R26*. In chimpanzee, two of the three chromosome 22 retrocopies show the same intron-derived insertion. Sequence data for the third chromosome 22 retrocopy in the chimpanzee is incomplete in this region because of a sequencing gap that spans sequences corresponding to the 3'-part of exon 1 and the 5'-part of exon 2. The third chimpanzee retrocopy is truncated by 1500 bp at the 3’-end of exon 4, and one of the other two chimpanzee chromosome 22 retrocopies is truncated at the 5’-end. 

Apart from the retrocopies in the *RB1* gene and on chromosome 22 in human and chimpanzee, the Blat search revealed additional retrocopies only on chimpanzee chromosome 8 and marmoset chromosome 4. Both consist of complete exons 1, 2, 3 and 4. The retrocopy on chimpanzee chromosome 8 is located in a gene desert, whereas the retrocopy on marmoset chromosome 4 is located in a region that corresponds to the major histocompatibility complex class I region on human chromosome 6 and harbours several genes, including *HLA-E*, *HLA-L*, *TRIM39* and *RPP21*. 

### *PPP1R26* and its retrocopies contain CpG islands

Human, chimpanzee and orangutan have a large (>300 bp) CpG island at the promoter region of the *PPP1R26* gene (UCSC CpG island track) [11]. Human and orangutan have four additional small CpG islands in exon 4 of *PPP1R26*, whereas chimpanzee and marmoset have one small and one large CpG island, and rhesus macaque has two small and one large CpG island in this region. Galago has no CpG islands in exon 4 (Figure 1). We have not included tarsier to Figure 1 as only part of exon 4 of *PPP1R26* was detected and no methylation analysis was conducted (no material was available).

The retrocopy in *RB1* intron 2, *PPP1R26P1*, is 5'-truncated and has no CpG island in what is left from exon 1. However, the region of *PPP1R26P1* that corresponds to exon 4 of the parental *PPP1R26* gene has two large CpG islands in all Anthropoidea that we studied. Rhesus macaque has one additional and marmoset has two additional small CpG islands in this region. 

The four retrocopies on human chromosome 22 all have a large CpG island in the sequence corresponding to exon 1 and no CpG island in the sequence corresponding to exon 4. The two retrocopies on chimpanzee chromosome 22 containing a full length exon 1 have a CpG island in this region, although the true extent of the CpG island in one of these retrocopies might be underestimated because of a sequencing gap in the available genome data. Both retrocopies with full length exon 4 have one small CpG island near the 3'-end.

The retrocopies on chimpanzee chromosome 8 and marmoset chromosome 4 have a large CpG island in exon 1. The sequences derived from exon 4 of the parental gene show two large CpG islands in marmoset, and one large and one small CpG island in chimpanzee. However, as the small CpG island (CpG 18) in the chimpanzee is next to a sequencing gap in the genome database, this CpG island could in fact be larger than annotated. 

### CpG islands in *PPP1R26* and its retrocopies have individual methylation patterns

**Figure 2:**
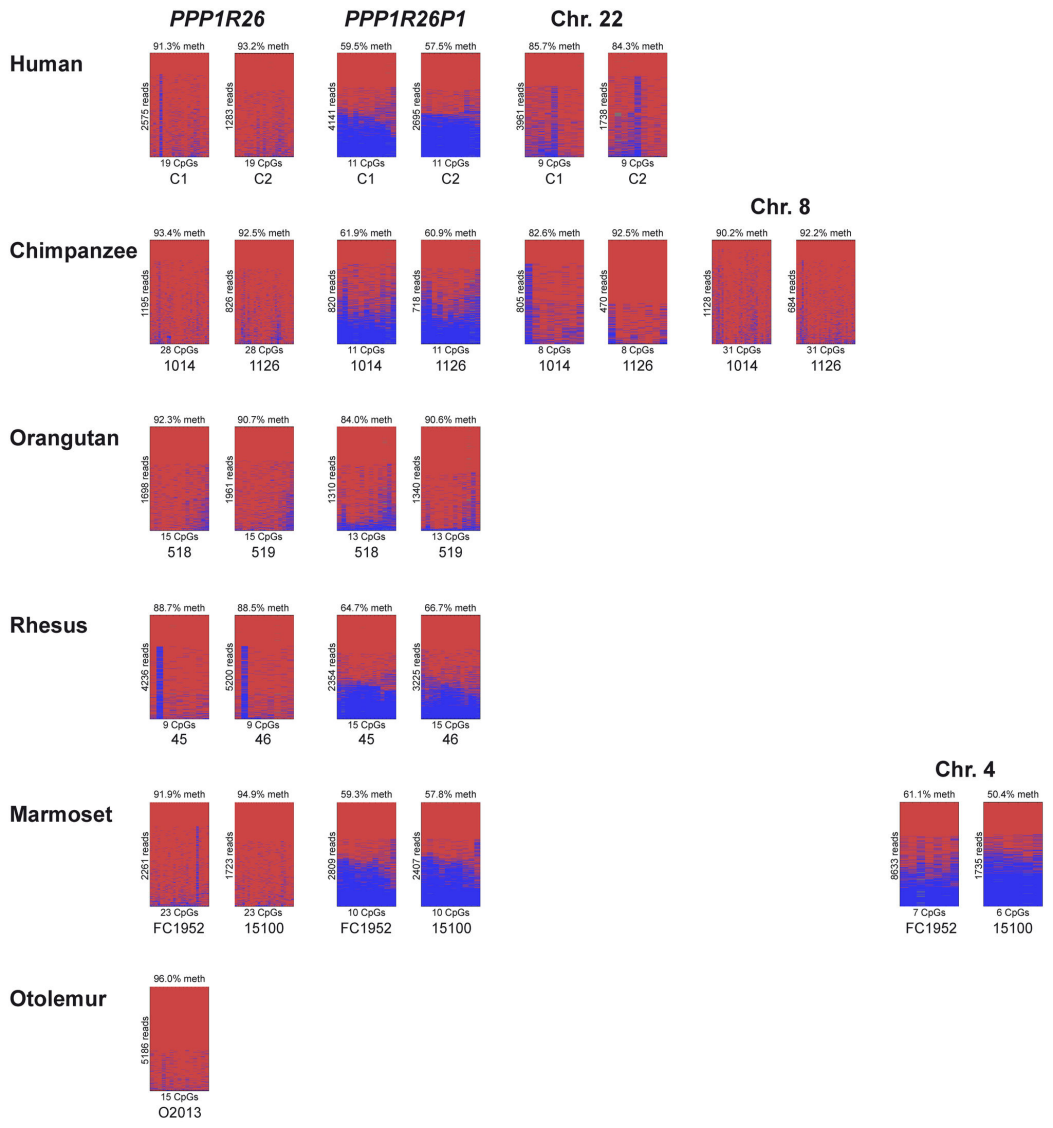
Methylation status of CpG islands in *PPP1R26* and its retrocopies. The CpG islands in the 5’-part of exon 4 studied for *PPP1R26* and its retrocopies on chromosome 22 and 8 are fully methylated. Only the retrocopy in intron 2 of the RB1 gene (orangutan shows differential methylation only at some CpG sites) and the additional retrocopy on chromosome 4 in marmoset show differential methylation. red, methylated; blue, unmethylated. Blood sample IDs are given under the images (C1, C2 - human; 1014, 1126 - chimpanzee; 518, 519 - orangutan; 45, 46 - rhesus macaque; FC49, 15100 - marmoset; O2013 - galago).

We used deep bisulfite sequencing to study the methylation patterns of the CpG islands in exon 4 of *PPP1R26* and in its retrocopies in primates (human, chimpanzee, rhesus macaque, orangutan, marmoset and galago; Figure 2). Two individual samples were analysed for each primate species, except for galago, for which only one sample was available. Detailed data and the mean methylation levels of each CpG island are shown in Figure 2. In figure 1, a pie chart indicates the average methylation at each CpG island analysed. In all primates studied, the CpG islands in *PPP1R26* exon 4 showed a methylation level of around 90% (Figure 1 pie charts, Figure 2). There are no CpG islands annotated in exon 4 in galago, so we studied the methylation of the region that corresponds by location to the CpG island analysed for *PPP1R26*. 

**Figure 3:**
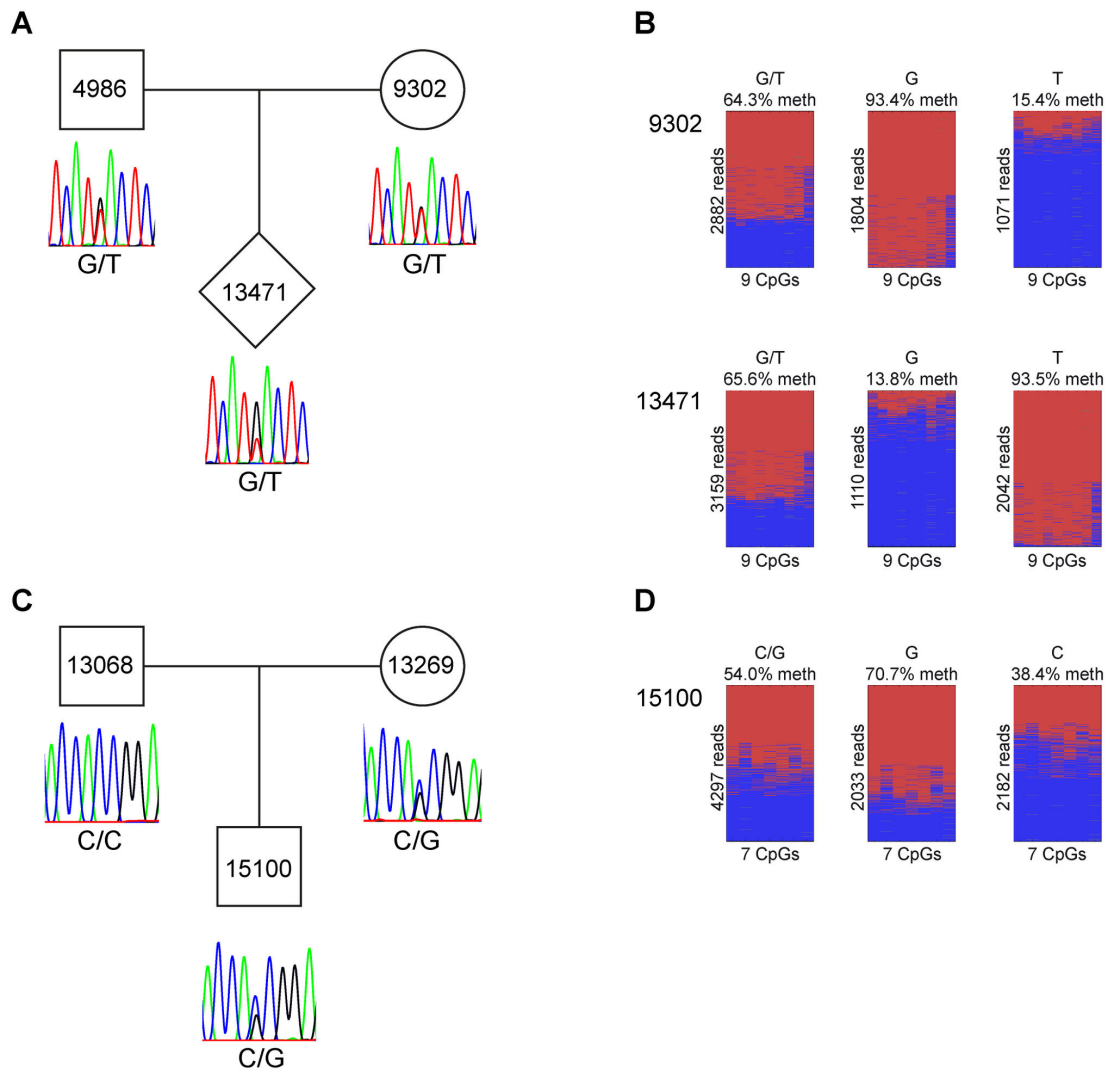
Analysis of parent-of-origin-specific methylation of the retrocopy in the *RB1* gene. (A) All rhesus macaque family members are heterozygous (G/T), thus, the parental origin of the alleles cannot be resolved. (B) One allele is methylated and the other allele is unmethylated in both individuals studied. (C) The offspring of the marmoset family carries an informative SNP (C/G). (D) The degree of methylation of the supposed maternal allele is 72%, whereas the supposed paternal allele shows 39% methylation. A, C Sanger sequencing of genomic DNA; B, D deep bisulfite sequencing.

The large CpG island in the exon 4 region of *PPP1R26P1* showed methylated and unmethylated sequences in all primates studied (Figure 1 pie charts, Figure 2). The degree of methylation was about 60% in all species except orangutan, which showed differential methylation only at some CpG sites whereas adjacent regions were highly methylated. Separating the orangutan alleles revealed that one allele is completely methylated at these sites whereas the other allele is 56% methylated (Figure S3). Previously, we have shown that the methylation at this CpG island is parent-of-origin-specific in human as the paternal allele is unmethylated and the maternal allele is methylated [10]. Analysis of members of a rhesus macaque family heterozygous for a SNP (G/T) in this region revealed that one allele is methylated and the other allele is unmethylated in rhesus as well (Figure 3A and B). However, the parental origin of the alleles cannot be resolved as all family members are heterozygous. In a marmoset family with an informative SNP (C/G), average methylation of the two alleles was 72% and 39% (Figure 3C and D). Determination of the parental origin of the alleles is not feasible due to the high rates of genetic microchimerism in marmosets [12]. In addition to the differentially methylated CpG island (CpG 85) that leads to imprinted *RB1* expression in human, the region of *PPP1R26P1* in Anthropoidea that is derived from exon 4 of the parental gene contains another CpG island (CpG 42 in the human). We analysed 15 CpGs within human CpG 42 and found full methylation (Figure 1, Figure S4).

As there are no CpG islands annotated in the first part of exon 4 of the retrocopies on chromosome 22 in human and chimpanzee, we analysed the methylation of the region corresponding to CpG 85 in human. We could not design specific primers for each of the four retrocopies on human chromosome 22 as they have a very high sequence identity. Therefore, we amplified all copies at once for the methylation analysis. We only obtained reads from methylated sequences (Figure 1, Figure 2). Methylation analysis of chimpanzee chromosome 22 retrocopies showed that the region corresponding to CpG 85 in human is fully methylated in each retrocopy (Figure 1, Figure 2 and Figure S5). The same result was obtained for the chimpanzee retrocopy on chromosome 8 (Figure 1, Figure 2).

**Figure 4:**
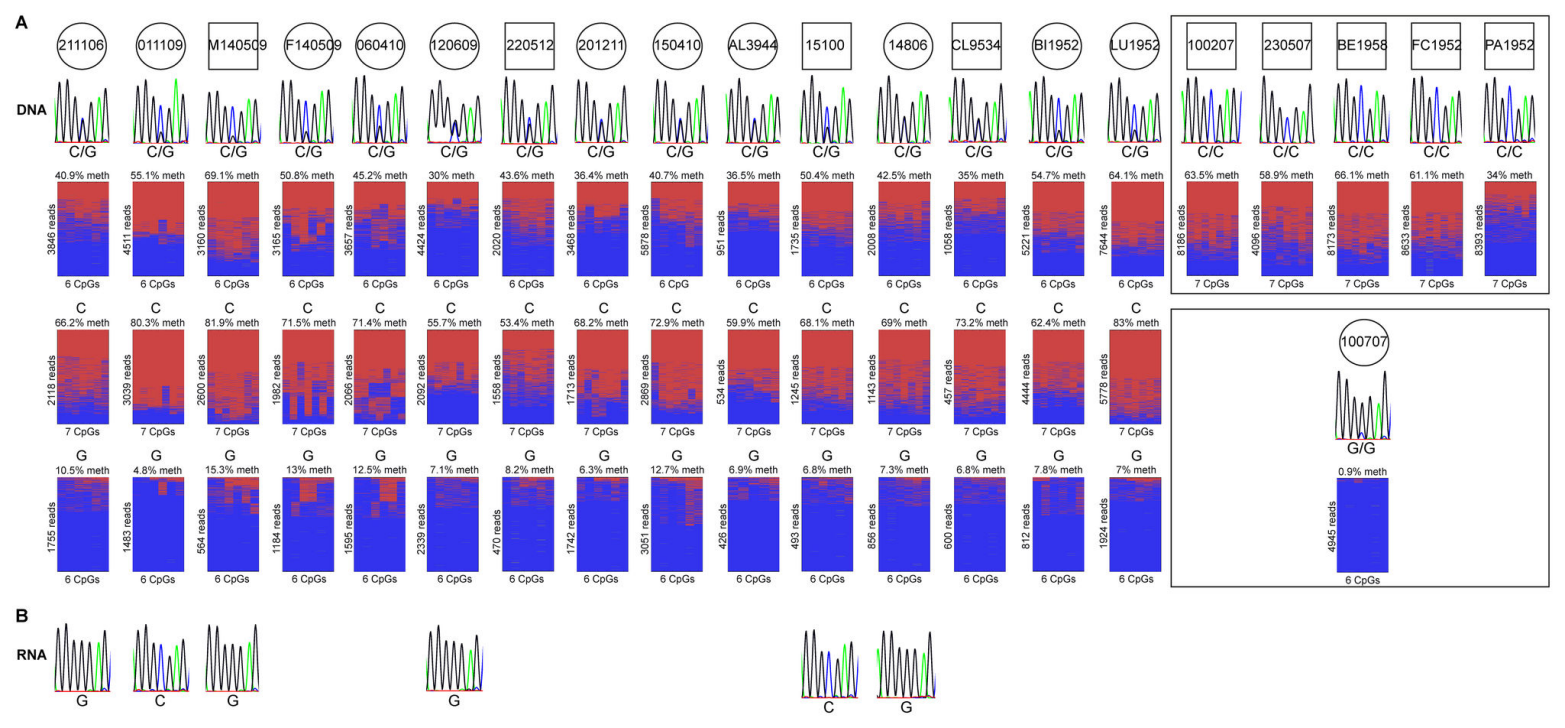
Allele-specific methylation and independent monoallelic expression of the retrocopy on chromosome 4 in marmoset. (A) A SNP (C/G) was used to distinguish the alleles. The mean methylation in the 15 heterozygous individuals was 30-69%, the G allele showed 5-15% methylation and the C allele showed 53-83% methylation. The 5 individuals homozygous for the C allele showed a mean methylation level of 34-66%. The individual homozygous for the G allele (100707) showed no methylation. Thus, the methylation pattern of the retrocopy on marmoset chromosome 4 is allele-specific. red, methylated; blue, unmethylated. (B) By RT-PCR and sequencing, a transcript specific for this retrocopy was identified in 6 heterozygous individuals (no RNA was available from the other heterozygous individuals). This transcript is monoallelically expressed and its expression is not regulated by DNA methylation as transcripts from methylated or unmethylated alleles were obtained.

Analysis of the CpG island in exon 4 of the retrocopy on marmoset chromosome 4 showed reads from methylated and unmethylated sequences. We analysed samples from two individuals and obtained methylations levels of 61% and 50% (Figure 2). An informative SNP (C/G) was used to check whether methylation of this retrocopy is allele-specific and to distinguish alleles of 15 individuals (Figure 4A). The mean methylation level in these 15 individuals was 30-69%. All 15 G alleles present in these individuals were almost unmethylated (5-15% methylation) and all 15 C alleles showed a methylation level of 53-83% (Figure 4A). Five individuals homozygous for the C allele showed a methylation level of 34-66% and one individual homozygous for the G allele was unmethylated (1%). Apparently, the methylation pattern of the retrocopy on marmoset chromosome 4 is allele-specific. 

### The retrocopy on marmoset chromosome 4 is monoallelically transcribed

We analysed marmoset chromosome 4 more closely by RT-PCR and sequencing and identified a transcript specific for this retrocopy (Figure 4B, Figure S6). Furthermore, an informative SNP showed that this transcript is monoallelically expressed (Figure 4B, Figure S6). However, expression does not appear to be methylation-specific because transcripts were obtained from the methylated (2 individuals) or unmethylated (4 individuals) allele (no RNA was available from the other heterozygous individuals). Again, a statement on the parental origin of the expressed allele is not possible because of microchimerism in marmoset [12]. We conducted 3’-RACE and 5’-RACE to determine the transcription start site and the total length of the transcript detected by a non-intron-spanning RT-PCR, but we did not obtain specific products.

### *PPP1R26P1* serves as a promoter for an alternative *RB1* transcript in rhesus macaque

**Figure 5:**
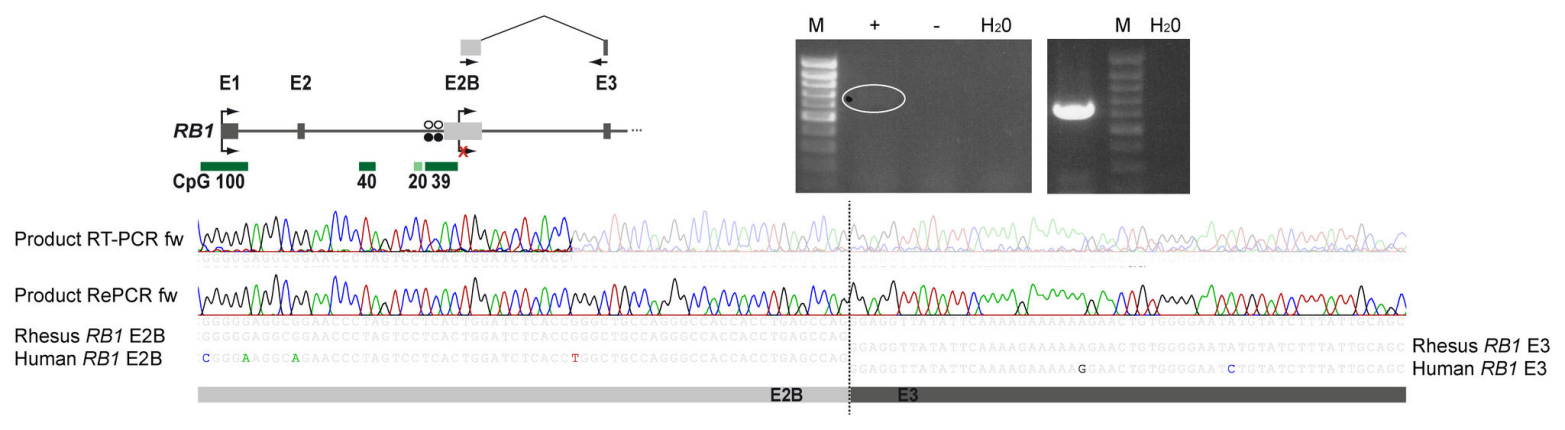
Expression of the alternative *RB1* transcript in rhesus macaque. By exon-connection RT-PCR of CpG 39 and exon 3 of the rhesus macaque RB1 gene an alternative RB1 transcript was detected. Top left: scheme of rhesus macaque RB1 and position of the primers. Top right: PCR products of RT-PCR and RePCR (reamplification of the RT-PCR product); as there was only a faint band visible after RT-PCR (white circle) RePCR was performed. Sequence analysis of the RT-PCR and RePCR product showed that it is specific for the 2B-transcript (bottom). M, MassRuler low range – Thermo Scientific , +, mRNA with reverse transcriptase (RT); -, mRNA without RT; H_2_O, negative control.

As we have previously shown that an alternative transcript is expressed in humans [10], we here used RT-PCR and sequencing to determine if alternative *RB1* transcripts are expressed in primates other than humans and if expression is monoallelic. RNA from blood was available from a rhesus macaque family and a marmoset family with individuals heterozygous for a SNP in *PPP1R26P1* (Figure 3A and C). Unfortunately, RT-PCR did not work on the RNAs from the rhesus macaque and marmoset individuals carrying the SNP. We could not detect expression of the 2B-transcript in marmoset although RT-PCRs with several different primers were tested. However, we obtained an RT-PCR product specific for the 2B-transcript for RNA from an uninformative rhesus macaque individual. Thus, the alternative *RB1* transcript is present in rhesus macaque, but it remains unsolved whether it is parent-of-origin-specifically expressed (Figure 5). 

## Discussion

Imprinted expression of the human *RB1* gene, a tumor suppressor gene with a plethora of functions [13], is the result of evolutionary change of a retrocopy of *PPP1R26* that was inserted into intron 2 of the *RB1* gene [10]. The comparison of the sequences of *PPP1R26* and its retrocopies in different primates performed in this study revealed that the *PPP1R26* gene is present in members of Strepsirrhini (galago) and Haplorrhini (Anthropoidea and tarsier). *PPP1R26P1*, however, was detected in all Anthropoidea species analysed here but not in tarsier or galago. This suggests that retrotransposition of *PPP1R26* is likely to have occurred before the split between Catarrhini and Platyrrhini. A further step in the evolution of *PPP1R26P1* in Anthropoidea is an insertion of an ~300 bp Alu repeat between exon 3 and exon 4 of *PPP1R26P1*. This repeat is missing in marmoset, and thus, was transposed into this region after the split between Platyrrhini and Catarrhini. Also after this split, but restricted to the clade that includes the marmoset, is a duplication of an ~550 bp region of exon 4 of *PPP1R26P1*. 

Imprinted expression of the human *RB1* gene is linked to differential expression from a promoter within a differentially methylated CpG island, CpG 85, located in the part of *PPP1R26P1* that is derived from exon 4 of the parental gene. All species known to have *PPP1R26P1* also have a CpG island that corresponds to CpG 85 in human. It is likely that a CpG-rich progenitor region of this CpG island was already present in the retrocopy that originally retrotransposed into the *RB1* gene, because in species with *PPP1R26P1* all extant orthologs of *PPP1R26* have one or two CpG islands in the 5’-part of exon 4, albeit of varying size. Large CpG islands (>300 bp) are also present in *PPP1R26* retrocopies on chimpanzee chromosome 8 and marmoset chromosome 4, further supporting that the parental *PPP1R26* gene contained a CpG island in the 5’-part of exon 4. 

In all primate species that we analysed, the CpG island in exon 4 of *PPP1R26* is methylated (Figure 1 pie charts, Figure 2), making it prone to CpG-loss by deamination. But as this region is fully contained in the open reading frame of *PPP1R26*, purifying selection can contribute to stabilization of its CpG content. The mechanisms that stabilize the CpG content in the CpG island in *PPP1R26P1* are less obvious. One possible factor is differential methylation of the human CpG 85 in *PPP1R26P1* and its homologues in the other primate species tested here. We have no explanation why the orangutan shows differential methylation only at a few CpG sites in this CpG island. In the marmoset individual carrying a SNP (C/G), the supposed maternal and paternal alleles showed a degree of methylation of 71% and 38%, respectively. This does not reveal a clear parent-of-origin-specific methylation pattern, which might be due to mircochimerism [12]. Although all members of the analysed rhesus macaque family are heterozygous and the parental origin of the alleles remains unresolved, it is likely that the methylation in rhesus macaque is parent-of-origin-specific like in human, as the detected monoallelic methylation is not allele-specific (Figure 3). Furthermore, it is plausible that the analysed CpG island in rhesus macaque is paternally unmethylated and maternally methylated like in human. Regions with differentially methylated CpGs and lower methylation in the male germ line are under lower mutational pressure due to methylation-coupled deamination compared to regions with lower methylation in the female germ line [14]. Moreover, it has been shown that the rate of CpG loss through non-deamination substitutions is also lower than expected in differentially methylated regions that serve as imprinting control regions [15]. 

We have shown in human that the *PPP1R26P1* region that was derived from exon 4 of *PPP1R26* has evolved into a promoter and an initial exon of an alternative *RB1* transcript that is expressed from the unmethylated allele at CpG 85 [10]. This transcript was also detected in rhesus macaque, suggesting that *PPP1R26P1* gained this function prior to the split of human and rhesus macaque. We attempted RT-PCR detection of this transcript in marmoset with different primers but obtained no product. We cannot exclude that an alternative *RB1* transcript is present in marmoset that we failed to detect for technical reasons only. Alternatively, such a transcript is not expressed in marmoset or at levels below the sensitivity of our approach. 

The *PPP1R26P1* in Catarrhini (human, chimpanzee, orangutan, rhesus macaque) have an insertion of an Alu repeat located 3’ relative to the start and direction of the alternative transcript. This repeat is not present in marmoset *PPP1R26P1*. It has been shown that Alu sequences inserted in the vicinity of promoters can enhance transcription rates [16]. Moreover, this repeat may also contribute to parent-of-origin-specific methylation of CpG 85 and its homologues, as Rubin and colleagues reported that Alu repeats are differentially methylated in primate germ cells [17]. They found hypomethylated and hypermethylated states in sperm and oocytes, respectively. This fits to our findings for the human Alu element in *PPP1R26P1*, which was completely unmethylated in sperm (Figure S7A). In blood however it showed a methylated state (Figure S7B). In the male germ line, Alu elements are selectively protected from DNA methylation by a specific Alu binding protein [18]. As hypomethylation in sperm does not affect all Alu sequences, there must be additional factors such as transcription, chromosomal location and nucleotide environment that may initialize and/or stabilize the methylation state [17,19]. Hellmann-Blumberg et al. showed that the methylation status of Alu elements varies in different tissues – Alus hypomethylated in sperm were completely methylated in spleen, fitting to our findings in human blood (Figure S7B) [20]. It is conceivable that the Alu element contributes to the establishment of the imprint but not to its maintenance. Apparently, the Alu element becomes methylated during differentiation when the specific Alu binding protein that provides the protection against methylation is not present. Possibly once the imprint is set by the Alu element and transcription takes place, the state of the imprint is maintained by transcription or rather the binding of transcription factors.

Using a SNP, we could show that the methylation of the retrocopy on marmoset chromosome 4, which consists of full exon 1-4 and no Alu insertion, is allele-specific. In this specific case microchimerism is irrelevant because the parental origin would not have an impact on DNA methylation. The marmoset homozygous for the G allele showed no methylation, whereas all marmosets homozygous for the C allele showed 34-66% methylation. Heterozygous marmosets had an almost unmethylated G allele (5-15% methylation) and 53-83% methylation of the C allele. Expression analysis revealed a transcript specific for this retrocopy that is monoallelically expressed but not in a methylation-specific manner. Due to the possibility of microchimerism in marmosets it is not possible to say whether the expression is parent-of-origin-specific [12]. Thus, the retrocopy on chromosome 4 in marmoset has allele-specific methylation and is monoallelically expressed, but it is not imprinted. It appears that this particular locus shows random monoallelic expression. The region that includes the retrocopy on chromosome 4 in marmoset corresponds to human chromosome 6 and contains several genes of the immune system, such as *HLA-E* and *TRIM39*. Several genes in human and mouse that are autosomal monoallelically expressed have functions in the immune or nervous system [21,22,23]. During differentiation, one allele is randomly silenced while the other allele remains active. This monoallelic state is then stably maintained across cell generations [24]. The expression observed at the retrocopy on chromosome 4 in marmoset could be an example for autosomal monoallelic expression, but it remains unclear whether this retrocopy has any contribution to this.

The retrocopies of *PPP1R26* on chromosome 22 in human and chimpanzee are located intergenically. Assuming that the retrocopies had a CpG island at the time of insertion, all retrocopies underwent rapid decay of their CpG content and show full methylation at the CpG dinucleotides that remained. It appears that the genomic environment in which these retrocopies were placed provided no portal of entry for remodelling of gene regulation. In contrast, the retrocopies inserted in or near genes gained differential methylation and regulatory functions, which in turn recruited the evolutionary forces that helped to maintain their high CpG content. Thus, our data supports the concept that genomic imprinting is based on host defence mechanisms, and that the epigenetic fate of a *PPP1R26* retrocopy after integration depends on the DNA sequence and selective forces at the integration site [25,26,27,28]. 

## Materials & Methods

### Ethics statement

Human blood samples from blood donors were obtained after written informed consent and were anonymised. The study was approved by the ethics committee of the University Duisburg-Essen (10-4396).

Non-human primate samples: Blood sampling procedures were conducted at the German Primate Centre in Göttingen. The studies were performed in accordance with the German Animal Welfare Act (Tierschutzgesetz der Bundesrepublik Deutschland 25.05.1998). This includes supervising and advice by the institutional animal welfare officer and approval by the governmental veterinary authorities. The corresponding reference number of the approval for blood sampling is 33.9-425-05-10A102 given by LAVES (Lower Saxony State Office for Consumer Protection and Food Safety). The ongoing of the procedures were controlled and supervised by the local and regional veterinary authorities, the veterinary staff and the animal welfare officer of the German Primate Centre. The animals are kept under conditions documented in the European Directive 2010/63/EU (directive on the protection of animals used for experimental and other scientific purposes) and the EU Recommendations 2007/526/EG (guidelines for the accommodation and care of animals used for experimental and other scientific purposes). These conditions are consistent with the regulations of the Guide for Care and Use of Laboratory Animals by the National Research Council (USA). The three Rs are considered using the 3Rs Guidelines for Primate Accommodation, Care and Use by the National Centre for the Replacement, Refinement and Reduction of Animals in Research (UK). 

The blood samples were obtained in combination with the annual health monitoring tests of the German Primate Center breeding colonies or in combination with necessary veterinary procedures in stock animals. None of the animals were euthanized. The procedures were performed in accordance with the described regulations of the local and regional veterinary authorities and under attention of the national and European animal welfare regulations (EU directive 2010/63 EU, German Animal Welfare Act). The institutional animal welfare officer, who has to agree to the procedure, was informed prior to the blood withdrawal. 

Below are details of housing conditions, enrichment and feeding.

Marmosets:

1 housing conditions
  ◦ indoor rooms with cages: 1qm and 2.50 m height for pairs and families with offspring; cages can be combined to allow larger groups
  ◦ indoor room: 14 to 18 qm, heated to 25 °C, humidity 60 %
  ◦ wood bedding on the ground
  ◦ enrichment with wood (bar, branches), ropes, sleeping boxes
2 feeding:
  ◦ two times per day in changing composition
  ◦ fruits, vegetables, special primate pellets, seeds
  ◦ cooked potatoes, eggs, rice, mealworms, grass hoppers, curd cheese, yoghurt
  ◦ Arabic gums
  ◦ water ad libitum

The German Primate Centre has a long standing experience in breeding, keeping and using non-human laboratory primates. Persons, who are carrying out procedures on animals, taking care for the animals or design the projects, have the authorisation by the veterinary authorities. An internal control system by the veterinary staff of the centre is established. This includes health monitoring, housing conditions, primate husbandry, care and environmental enrichment.

Additional non-human primate samples: Marmoset (*Callithrix jacchus*) DNA was obtained from blood samples provided by Stefan Schlatt, Centre of Reproductive Medicine and Andrology, Muenster. The blood samples were drawn related to experiments approved by the relevant authorities in accordance with the German Federal Law on the Care and Use of Laboratory Animals (LANUV North Rhine-Westphalia, Licence No. 84-02.05.20.12.0.18). The marmoset monkeys from our breeding colony were kept in pairs/families under a 12 h light: 12 h darkness regimen and fed food pellets from Altromin (Lage, Germany) composed for marmosets together with beef or chicken meat and a daily supplement of fresh fruits and vegetables. They had unlimited access to tap water. Housing and exercise conditions were identical for all animals. No animals were sacrificed in the frame of this study. Blood samples (0.2 ml) were taken from the Vena femoralis. Sampling was performed without sedation by manual fixation by experienced animal caretakers, a method approved by the local authorities and proved to be less stress-causing than prior sedation of animals. Every animal was rewarded flour worms after blood sampling. The LANUV North Rhine-Westphalia, Licence No. 84-02.05.20.12.0.18 refers to the approval for blood sampling by the Ministry of Environment of North Rhine-Westphalia, Germany.

### *In silico* analysis

The BLAT tool of the UCSC genome browser was used for *in silico* analyses (human - *Homo sapiens*, hg19; chimpanzee - *Pan troglodytes*, panTro4; orangutan - *Pongo abelii*, ponAbe2; rhesus macaque - *Macaca mulatta*, rheMac3; marmoset - *Callithrix jacchus*, calJac3; tarsier - *Tarsius syrichta*, tarSyr1; galago - *Otolemur garnettii*, otoGar3). We also performed blastn which has more relaxed parameters and still ended up with no retrocopy of *PPP1R26* in galago or tarsier. The query sequence used for all searches was the mRNA sequence of *PPP1R26* (NM_014811). We also used Progressive Mauve to align the intron 2 regions of *RB1* of tarsier, galago, human and marmoset and again obtained the same results.

### Bisulfite treatment

Bisulfite treatment was carried out using the EZ DNA Methylation-Gold Kit (Zymo Research Europe, Freiburg, Germany) according to the manufacturer’s protocol.

### Deep bisulfite sequencing

Generation of bisulfite amplicon libraries, sample preparation and sequencing on the Roche 454 GS junior system was carried out as previously described [29]. Primer sequences are given in Table S1.

For data analysis, we used the Python-based amplikyzer software developed in-house [unpublished, available under the open source MIT License at https://code.google.com/p/amplikyzer/ ].

### Genomic sequence analysis

Primers designed for *RB1* (rhesus macaque, marmoset) and *PPP1R26P1* on marmoset chromosome 4 are listed in Table S1. For each PCR, 100 ng genomic DNA was used in a total volume of 25 µl. PCR conditions were as follows (for Tm=X see Table S1): 95°C for 10 min; 35 cycles of 95°C for 20 s, X°C for 30 s, 72°C for 1 min; 72°C for 7 min. The PCR products were purified by MultiScreen Filtration (Millipore, Billerica, MA, USA). The sequence reactions were performed with Big Dye Terminators (BigDye Terminator v1.1 Cycle Sequencing Kit, Applied Biosystems, Foster City, CA, USA) and the cycle sequencing procedure. Reaction products were analysed with an ABI 3100 automatic capillary Genetic Analyzer and Sequencing Analysis software (Applied Biosystems, Foster City, CA, USA).

### Expression analysis

RT-PCRs were performed with the GeneAmp RNA PCR Kit (Applied Biosystems, Foster City, CA, USA). Total RNA from blood was reverse transcribed with random hexamers. For amplification, the Advantage cDNA Polymerase Mix (Clontech, Mountain View, CA, USA) and the Phusion Flash High Fidelity PCR Master Mix (Finnzymes, Espoo, Finland) were used. PCR products were checked on an agarose gel and purified by gel extraction (Wizard SV Gel and PCR Clean-Up System, Promega). The primers used for the different RT-PCRs are listed in Table S1. For exon connection PCR at the *RB1* locus in rhesus macaque, we designed primers where the forward primer anneals to the CpG 39 CpG island in the retrocopy in *RB1* intron 2 and the reverse primer anneals to exon 3 of the *RB1* gene. For amplification, we used the Phusion Flash High Fidelity PCR Master Mix (Finnzymes, Espoo, Finland). PCR conditions were as follows: 98°C for 10 sec; 35 cycles of 98°C for 1 sec, 61°C for 5 sec, 72°C for 15 sec; 72°C for 1 min. 

For the expression analysis at the retrocopy on chromosome 4 in the marmoset we designed primers located in the analysed CpG island and used the Advantage cDNA Polymerase Mix (Clontech, Mountain View, CA, USA) for amplification. PCR conditions were as follows: 95°C for 1 min; 5 cycles of 95°C for 20 sec, 68°C for 3 min; 35 cycles of 95°C for 20 sec, 66°C for 3 min; 5 cycles of 95°C for 20 sec, 60°C for 3 min; 68°C for 3 min.

## Supporting Information

Table S1 **Primer sequences.**
(DOCX)

Figure S1 **Quantitative PCR analysis of *PPP1R26P1* in orangutan.** The normalized ratios of the target locus and the reference locus for each sample are shown (error bars = normalized ratio error; for the analysis the LightCycler 480 Software was used). *TRPS1* was used as a reference locus and rhesus macaque was used as a reference genome as it only contains one *PPP1R26P1* copy. The data show that there is only one copy of *PPP1R26P1* present in the orangutan genome. Rhe 42, Rhe 46, Rhe 48 – rhesus macaque samples; OU 518, OU 519 – orangutan samples.
(TIF)

Figure S2 **Progressive Mauve alignments.** In the upper part the alignment of the human *RB1* intron 2 region and the tarsier *RB1* intron 2 region is shown. Below the alignment of the human *RB1* intron 2 region and the Otolemur *RB1* intron 2 region is shown. For both tarsier and Otolemur the neighbouring sequences of *PPP1R26P1* are present but *PPP1R26P1* itself is clearly absent. Human hg19, chr13:48880916-48917350_plus strand; tarsier syrTar1, scaffold_292:1-45070_minus strand; Otolemur otoGar3, GL873625:966173-991826_plus strand.
(TIF)

Figure S3 **Methylation status of CpG 81 in orangutan *PPP1R26P1*.** The alleles for individual 519 were separated using a SNP (C/G). The degree of methylation for each CpG site is shown. On the left: alleles, on the right: mean methylation over the four CpG sites and number of reads.
(TIF)

Figure S4 **Methylation status of CpG 42 in human *PPP1R26P1*.** All analysed samples are fully methylated. red, methylated; blue, unmethylated. Blood sample IDs are given under the images (C1, C2 and C3 - human).
(TIF)

Figure S5 **Methylation status of the three retrocopies on chimpanzee chromosome 22.** All three retrocopies on chimpanzee chromosome 22 are methylated. red, methylated; blue, unmethylated. Samples IDs are given under the images (1014, 1126 - chimpanzee).
(TIF)

Figure S6 **Methylation and expression analysis of the retrocopy on marmoset chromosome 4.** A SNP (C/G) was used to distinguish the alleles. In all analysed samples, the G allele showed 7% methylation and the C allele showed 56-68% methylation. The individual homozygous for the G allele (100707) showed no methylation. Thus, the methylation pattern of the retrocopy on marmoset chromosome 4 is allele-specific. red, methylated; blue, unmethylated. By RT-PCR and sequencing, a transcript specific for this retrocopy was identified. Based on the informative SNP (C/G), it could be shown that this transcript is monoallelically expressed. Moreover, the expression is not regulated by DNA methylation as transcripts from methylated or unmethylated alleles were obtained.
(TIF)

Figure S7 **Methylation status of the Alu element in *PPP1R26P1* of human sperm.** (A) The Alu element is completely unmethylated in sperm (1168-4 and 1167-33 - human) and (B) methylated in blood (C1, C2 - human). red, methylated; blue, unmethylated.
(TIF)
