# Supplementary material for: The Origin of the RB1 Imprint
Source: PLoS One. 2013 Nov 25;8(11):e81502. doi: 10.1371/journal.pone.0081502 (PMC3839921; doi:10.1371/journal.pone.0081502)
Supplement: Table S1 — Primer sequences. (DOCX) [file pone.0081502.s001.docx]

| **Primer name** | **Primer sequence (5‘-3‘)** | **Tm (°C)** | **Product size (bp)** | **Species** |
| --- | --- | --- | --- | --- |
| **Next generation bisulfite sequencing** (universal tags in **bold**) | | | |  |
| Human-RB1-Ftag | **CTTGCTTCCTGGCACGAG**TATTTGGATGGTTTTTTTAG | 53 | 223 | *Homo sapiens* |
| Human-RB1-RM13 | **CAGGAAACAGCTATGAC**AACAAAAAAACAAAAACAACC |  |  |  |
| Human-Chr9-Ftag | **CTTGCTTCCTGGCACGAG**GGATGGTTTTTTTAATGTTA | 48 | 304 |  |
| Human-Chr9-RM13 | **CAGGAAACAGCTATGAC**CCAAAAAAAATTTACCTTC |  |  |  |
| Human-Chr22-Ftag | **CTTGCTTCCTGGCACGAG**AATTTTATTTTTATTTTGTA | 46 | 234 |  |
| Human-Chr22-RM13 | **CAGGAAACAGCTATGAC**ACTACAAAAATAATACAC |  |  |  |
| Bis-CpG42-Ftag | **CTTGCTTCCTGGCACGAG**GAGGGTTTAGATTTTTATTG | 54 | 233 |  |
| Bis-CpG42-RM13-1 | **CAGGAAACAGCTATGAC**AACCCTAACCAAACAAC |  |  |  |
| Chimp-RB1-Ftag | **CTTGCTTCCTGGCACGAG**AGTATATTTGGATGGTTTTTTTAG | 51 | 227 | *Pan troglodytes* |
| Chimp-RB1-RM13 | **CAGGAAACAGCTATGAC**AACAAAAAAACAAAAACAACC |  |  |  |
| Chimp-Chr8u9-Ftag | **CTTGCTTCCTGGCACGAG**TTTTATTTTTATTTTGTG | 56 | 494 |  |
| Chimp-Chr9-RM13 | **CAGGAAACAGCTATGAC**TAAAATCCCACCAAAAACTAC |  |  |  |
| Chimp-Chr8-RM13 | **CAGGAAACAGCTATGAC**TTAAAATCCCACCAAAAACC |  |  |  |
| Chimp-Chr22.2-Ftag | **CTTGCTTCCTGGCACGAG**TATGATTTTGGATTTGAGGG | 56 | 328 |  |
| Chimp-Chr22-RM13 | **CAGGAAACAGCTATGAC**CCCAATCAACATAAACAAA |  |  |  |
| OU-RB1-Ftag | **CTTGCTTCCTGGCACGAG**TATATTTGGATGGTTTTTTTAG | 62 | 251 | *Pongo abelii* |
| RB1-RM13 | **CAGGAAACAGCTATGAC**AAACCTCAAATCCAAAATCAC |  |  |  |
| OU-Chr9-Ftag | **CTTGCTTCCTGGCACGAG**GGTATAGTTGGATGGTTTTTTTAA | 56 | 247 |  |
| OU-Chr9-RM13 | **CAGGAAACAGCTATGAC**CAAATCCAAAATCACAACCAC |  |  |  |
| Rhe-RB1-Ftag-Z | **CTTGCTTCCTGGCACGAG**GGGATTTGTTGTTGTTTTA | 56 | 258 | *Macaca mulatta* |
| Rhe-RB1-RM13-Z | **CAGGAAACAGCTATGAC**AAAACCACAAACCCCTAC |  |  |  |
| Rhe-Chr15-Ftag-X | **CTTGCTTCCTGGCACGAG**ATATTAGTTTAGTAGATG | 48 | 212 |  |
| Rhe-Chr15-RM13-X | **CAGGAAACAGCTATGAC**CACTCAACAAAAAAAAACCC |  |  |  |
| Rhe-RB1-SNP-Ftag | **CTTGCTTCCTGGCACGAG**ATTTGGATGGTTTTTTTAG | 50 | 168 |  |
| Rhe-RB1-SNP-RM13 | **CAGGAAACAGCTATGAC**CACCACAAAATAAAAATAAAAT |  |  |  |
| M-Chr1-Ftag | **CTTGCTTCCTGGCACGAG**GTATTGTTTGTGTTGTGTGTA | 53 | 424 | *Callithrix jacchus* |
| M-Chr1-RM13 | **CAGGAAACAGCTATGAC**ATAAACACCCAAAAAAAACC |  |  |  |
| M-RB1-Ftag | **CTTGCTTCCTGGCACGAG**TATATTTGGATTGTTTTTTTAATG | 56 | 205 |  |
| M-RB1-RM13-1 | **CAGGAAACAGCTATGAC**TACAAAATCAACAACCCCTATCAC |  |  |  |
| neuBis-Mchr4-Ftag | **CTTGCTTCCTGGCACGAG**TAGAAGTAATATAGAATTAAAGTGGGATG | 60 | 183 |  |
| neuBis-Mchr4-RM13 | **CAGGAAACAGCTATGAC**ACAACTAAATAACCTCCTCRATACT |  |  |  |
| M-RB1-Ftag | **CTTGCTTCCTGGCACGAG**TATATTTGGATTGTTTTTTTAATG | 52 | 639 |  |
| M-RB1-SNP-RM13 | **CAGGAAACAGCTATGAC**TAAACCAAAACCAAATCATAAT |  |  |  |
| Oto-PPP1R26-Ftag | **CTTGCTTCCTGGCACGAG**AAAGAGTTGATTGTTAAGGGGGTAT | 58 | 396 | *Otolemur garnettii* |
| Oto-PPP1R26-RM13 | **CAGGAAACAGCTATGAC**CCTTCCTTATCTTCTCCAACTAATACA |  |  |  |
| **Genomic sequence analysis** | | | |  |
| Rhe-SNP-E2B-F2 | CGGAGCTTTGGTTTTCTTCG | 60 | 368 | *Macaca mulatta*/*RB1* |
| Rhe-SNP-E2B-R2 | TGGGGTCAAGAGGAGCG |  |  |  |
| M-RB1-E2B-F | GCCCTAGTTCTCTTGCGTGG | 60 | 450 | *Callithrix jacchus*/*RB1* |
| M-RB1-E2B-R1 | TCAGGTGGGGGCAGTAGATG |  |  |  |
| M-SNP-Chr4-F | TCCACGGTCACAACCACGTG | 68 | 709 | *Callithrix jacchus*/ retrocopy on chr4 |
| M-RNA-3-R | ACGTCCGGATTTCCTGCTCC |  |  |  |
| **Expression analysis** | | | |  |
| EC-Rhe-E2B-F2 | ACCTGGATGGCCTCCTTAG | 61 | 626 | *Macaca mulatta*/*RB1* |
| EC-Rhe-E3-R | AGCTCAGTAAAAGTGAATGGC |  |  |  |
| M-SNP-Chr4-F | TCCACGGTCACAACCACGTG | 66 | 709 | *Callithrix jacchus*/ retrocopy on chr 4 |
| M-RNA-3-R | ACGTCCGGATTTCCTGCTCC |  |  |  |

**Table S1.** Primer sequences.
