# Supplementary figures and images for: The Origin of the RB1 Imprint
Source: PLoS One. 2013 Nov 25;8(11):e81502. doi: 10.1371/journal.pone.0081502 (PMC3839921; doi:10.1371/journal.pone.0081502)

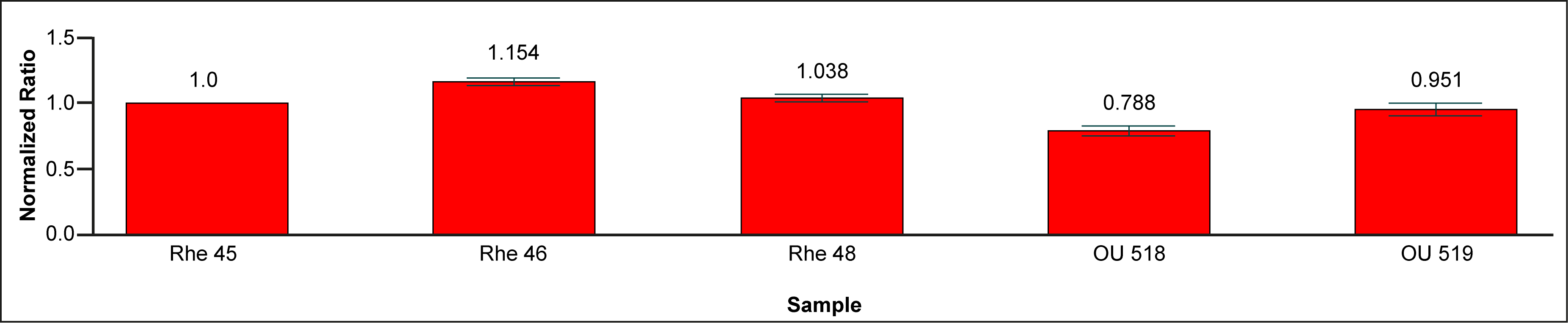

Supplement: Figure S1 — Quantitative PCR analysis of PPP1R26P1 in orangutan. The normalized ratios of the target locus and the reference locus for each sample are shown (error bars = normalized ratio error; for the analysis the LightCycler 480 Software was used). TRPS1 was used as a reference locus and rhesus macaque was used as a reference genome as it only contains one PPP1R26P1 copy. The data show that there is only one copy of PPP1R26P1 present in the orangutan genome. Rhe 42, Rhe 46, Rhe 48 – rhesus macaque samples; OU 518, OU 519 – orangutan samples. (TIF) [file pone.0081502.s002.tif]

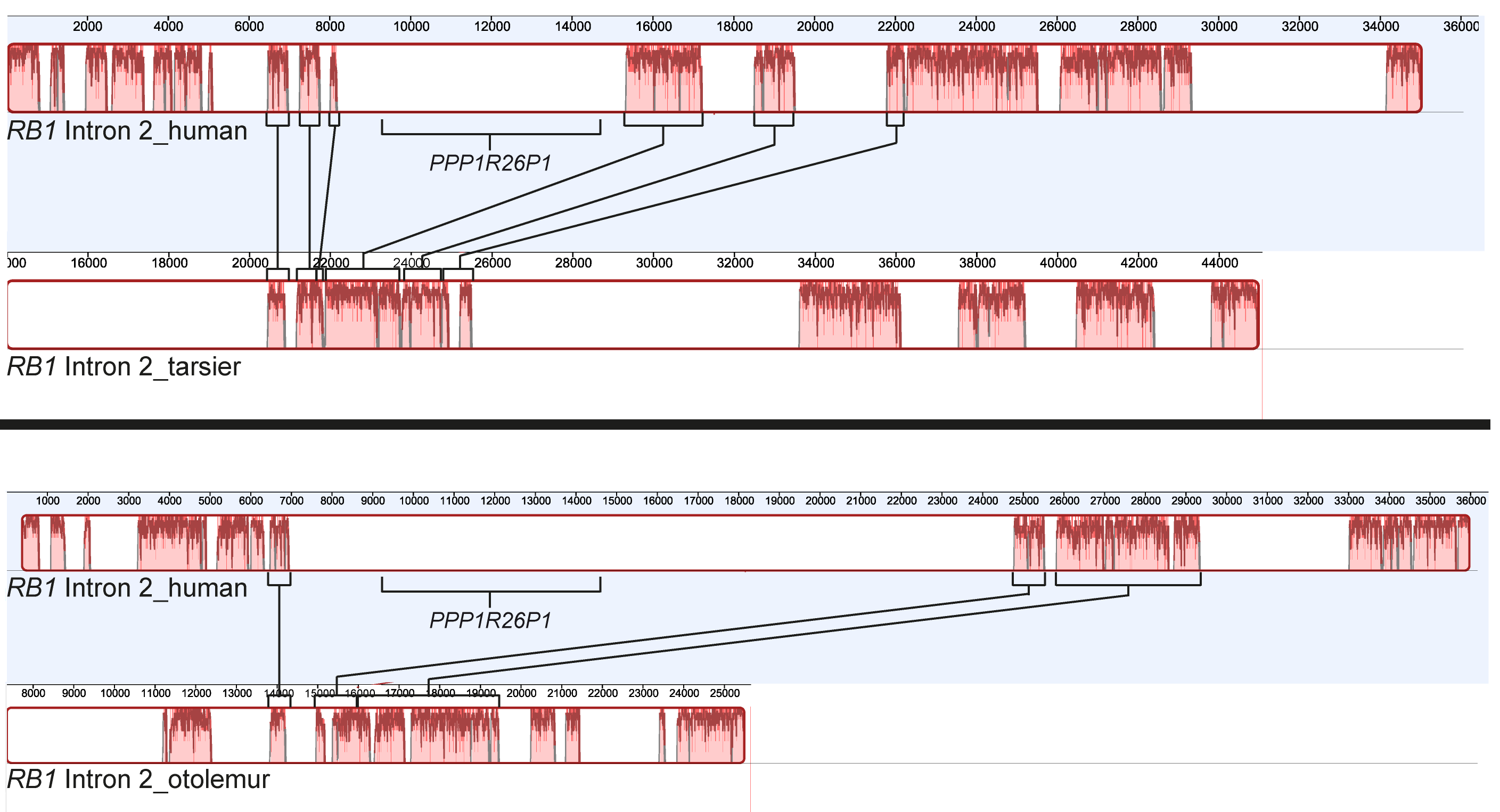

Supplement: Figure S2 — Progressive Mauve alignments. In the upper part the alignment of the human RB1 intron 2 region and the tarsier RB1 intron 2 region is shown. Below the alignment of the human RB1 intron 2 region and the Otolemur RB1 intron 2 region is shown. For both tarsier and Otolemur the neighbouring sequences of PPP1R26P1 are present but PPP1R26P1 itself is clearly absent. Human hg19, chr13:48880916-48917350_plus strand; tarsier syrTar1, scaffold_292:1-45070_minus strand; Otolemur otoGar3, GL873625:966173-991826_plus strand. (TIF) [file pone.0081502.s003.tif]

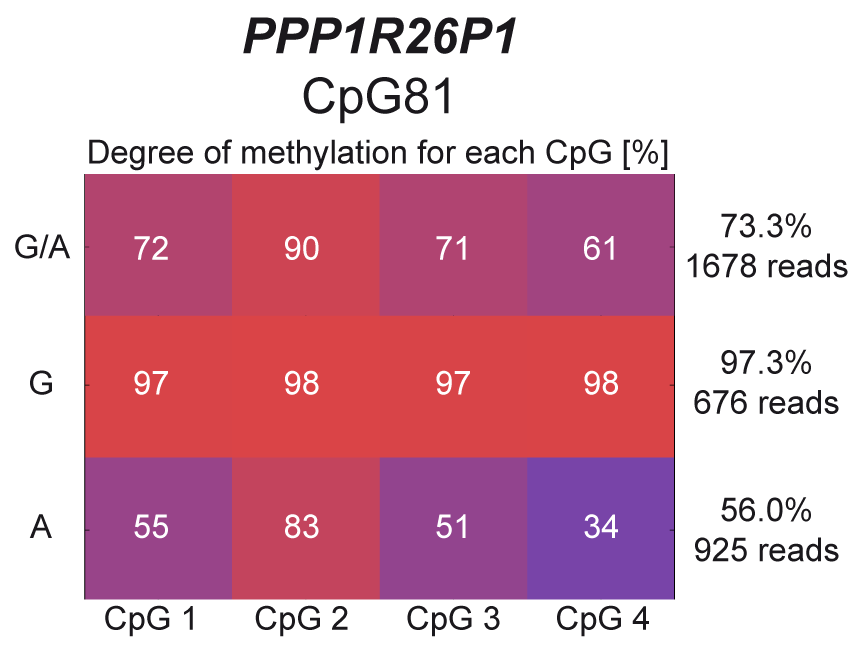

Supplement: Figure S3 — Methylation status of CpG 81 in orangutan PPP1R26P1. The alleles for individual 519 were separated using a SNP (C/G). The degree of methylation for each CpG site is shown. On the left: alleles, on the right: mean methylation over the four CpG sites and number of reads. (TIF) [file pone.0081502.s004.tif]

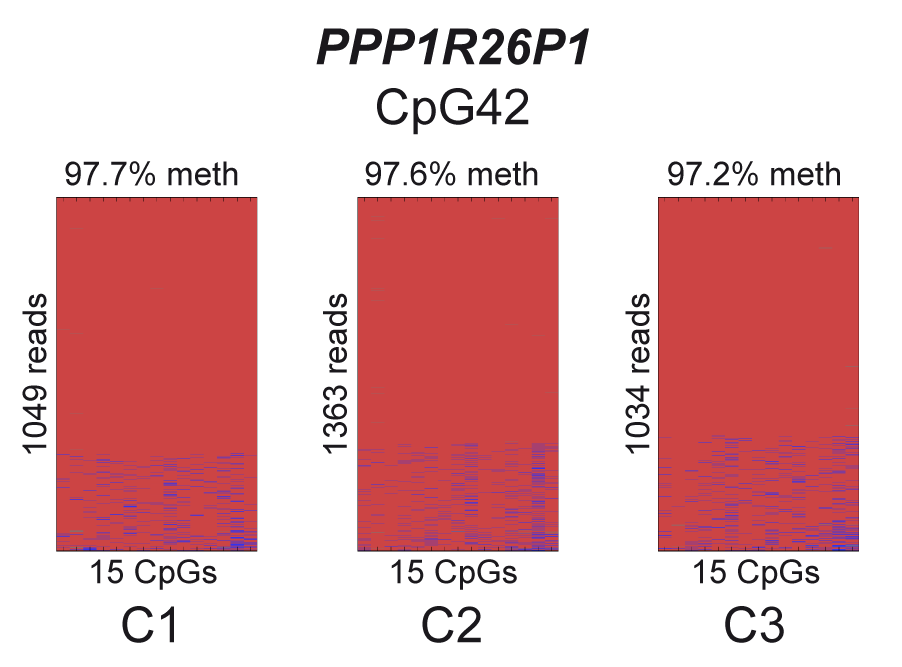

Supplement: Figure S4 — Methylation status of CpG 42 in human PPP1R26P1. All analysed samples are fully methylated. red, methylated; blue, unmethylated. Blood sample IDs are given under the images (C1, C2 and C3 - human). (TIF) [file pone.0081502.s005.tif]

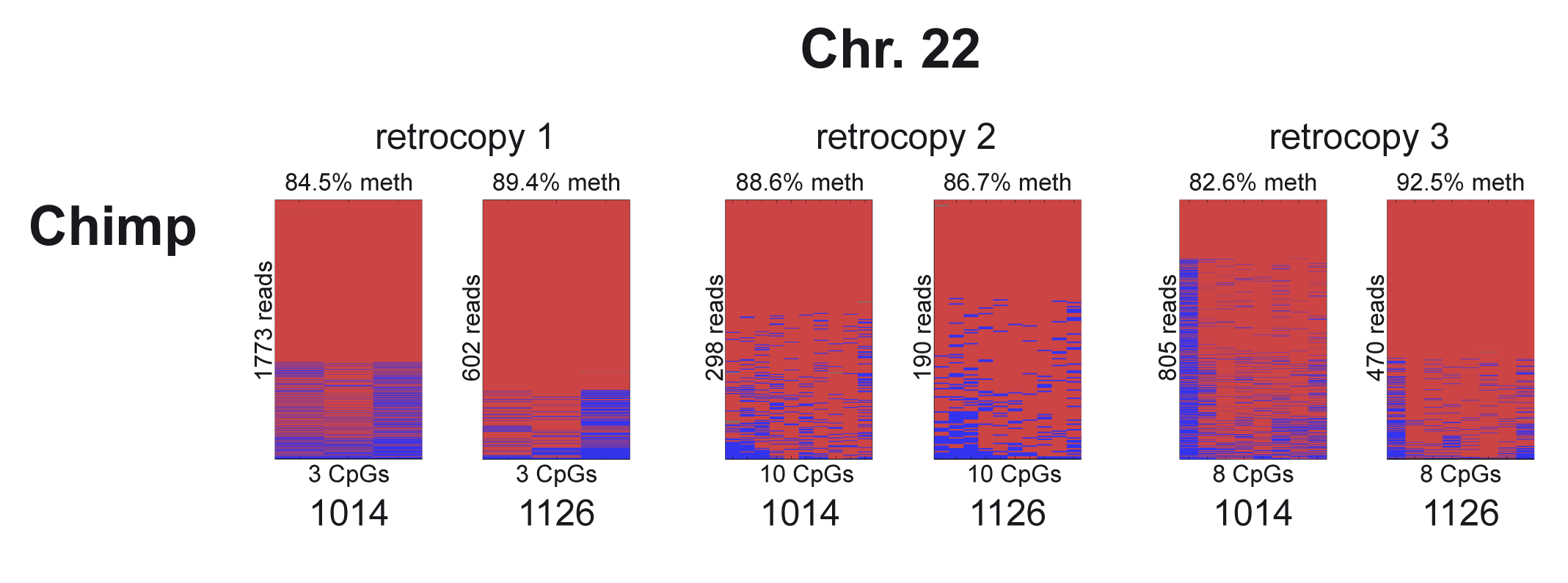

Supplement: Figure S5 — Methylation status of the three retrocopies on chimpanzee chromosome 22. All three retrocopies on chimpanzee chromosome 22 are methylated. red, methylated; blue, unmethylated. Samples IDs are given under the images (1014, 1126 - chimpanzee). (TIF) [file pone.0081502.s006.tif]

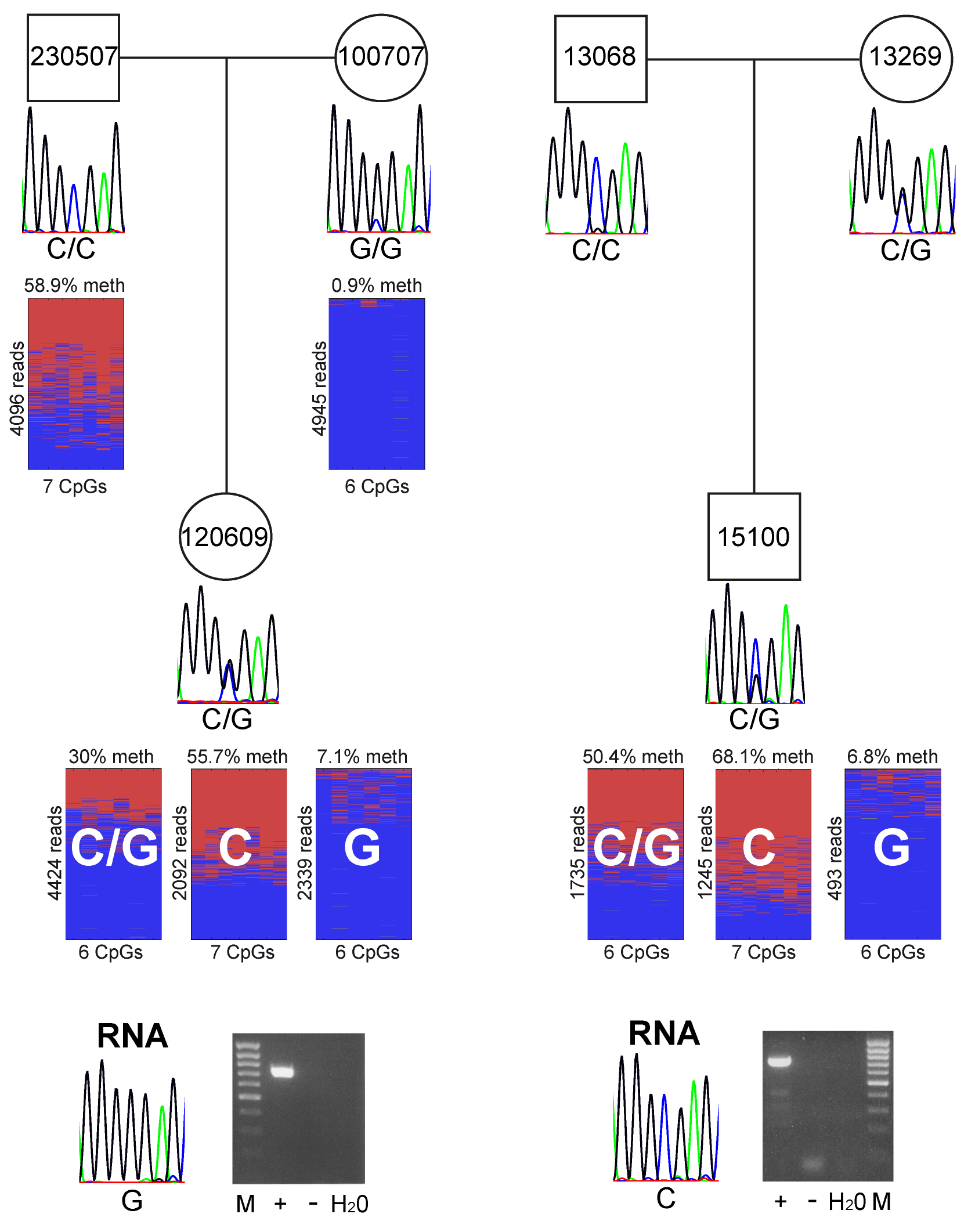

Supplement: Figure S6 — Methylation and expression analysis of the retrocopy on marmoset chromosome 4. A SNP (C/G) was used to distinguish the alleles. In all analysed samples, the G allele showed 7% methylation and the C allele showed 56-68% methylation. The individual homozygous for the G allele (100707) showed no methylation. Thus, the methylation pattern of the retrocopy on marmoset chromosome 4 is allele-specific. red, methylated; blue, unmethylated. By RT-PCR and sequencing, a transcript specific for this retrocopy was identified. Based on the informative SNP (C/G), it could be shown that this transcript is monoallelically expressed. Moreover, the expression is not regulated by DNA methylation as transcripts from methylated or unmethylated alleles were obtained. (TIF) [file pone.0081502.s007.tif]

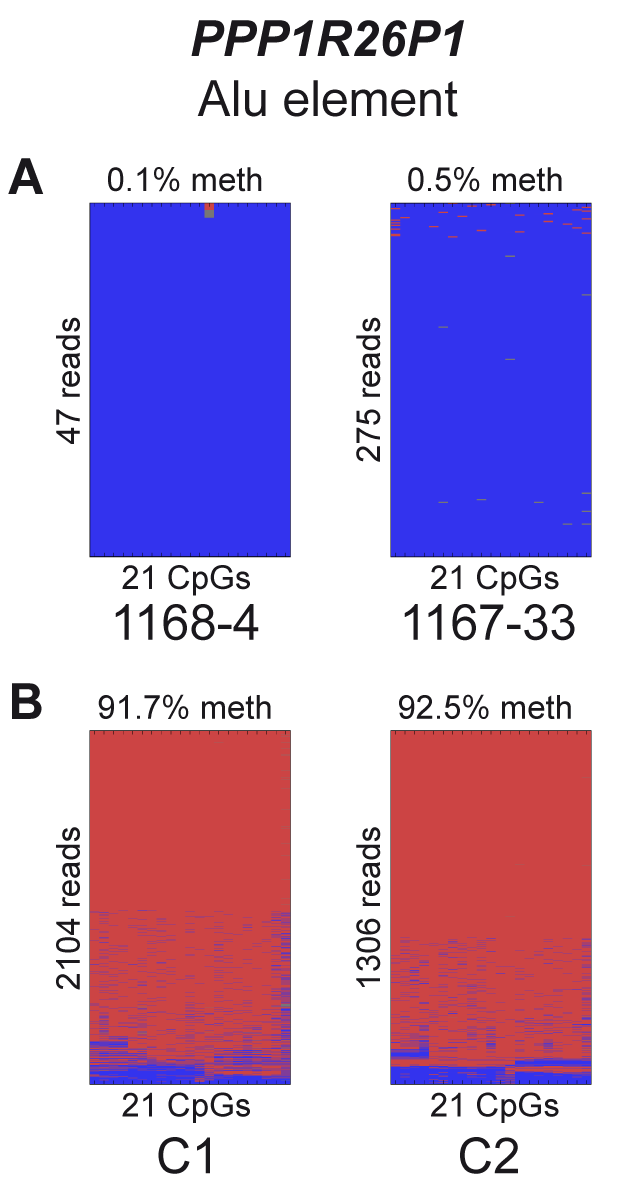

Supplement: Figure S7 — Methylation status of the Alu element in PPP1R26P1 of human sperm. (A) The Alu element is completely unmethylated in sperm (1168-4 and 1167-33 - human) and (B) methylated in blood (C1, C2 - human). red, methylated; blue, unmethylated. (TIF) [file pone.0081502.s008.tif]
